# Association of Adequacy of Broadband Internet Service With Access to Primary Care in the Veterans Health Administration Before and During the COVID-19 Pandemic

**DOI:** 10.1001/jamanetworkopen.2022.36524

**Published:** 2022-10-17

**Authors:** Amy M. J. O’Shea, Aaron Baum, Bjarni Haraldsson, Ariana Shahnazi, Matthew R. Augustine, Kailey Mulligan, Peter J. Kaboli

**Affiliations:** 1 Veterans Rural Health Resource Center–Iowa City, Veterans Affairs Office of Rural Health, and Center for Access and Delivery Research and Evaluation at the Iowa City Veterans Affairs Healthcare System, Iowa City, Iowa; 2 Department of Internal Medicine, University of Iowa Carver College of Medicine, Iowa City; 3 Department of Research and Development, Waymark, San Francisco, California; 4 Department of Health System Design and Global Health, Icahn School of Medicine at Mount Sinai, New York, New York; 5 New York Harbor Veterans Affairs Healthcare System, New York, New York; 6 Geriatric Research Education and Clinical Center, James J. Peters Veterans Affairs Medical Center, Bronx, New York; 7 Division of General Internal Medicine, Department of Medicine, Icahn School of Medicine at Mount Sinai, New York, New York; 8 Department of Biostatistics, University of Iowa College of Public Health, Iowa City

## Abstract

**Importance:**

Although telemedicine expanded rapidly during the COVID-19 pandemic and is widely available for primary care, required broadband internet speeds may limit access.

**Objective:**

To identify disparities in primary care access in the Veterans Health Administration based on the association between broadband availability and primary care visit modality.

**Design, Setting, and Participants:**

This cohort study used administrative data on veterans enrolled in Veterans Health Administration primary care to identify visits at 937 primary care clinics providing telemedicine and in-person clinical visits before the COVID-19 pandemic (October 1, 2016, to February 28, 2020) and after the onset of the pandemic (March 1, 2020, to June 30, 2021).

**Exposures:**

Federal Communications Commission–reported broadband availability was classified as inadequate (download speed, ≤25 MB/s; upload speed, ≤3 MB/s), adequate (download speed, ≥25 <100 MB/s; upload speed, ≥5 and <100 MB/s), or optimal (download and upload speeds, ≥100 MB/s) based on data reported at the census block by internet providers and was spatially merged to the latitude and longitude of each veteran’s home address using US Census Bureau shapefiles.

**Main Outcomes and Measures:**

All visits were coded as in-person or virtual (ie, telephone or video) and counted for each patient, quarterly by visit modality. Poisson models with Huber-White robust errors clustered at the census block estimated the association between a patient’s broadband availability category and the quarterly primary care visit count by visit type, adjusted for covariates.

**Results:**

In primary care, 6 995 545 veterans (91.8% men; mean [SD] age, 63.9 [17.2] years; 71.9% White; and 63.0% residing in an urban area) were seen. Adjusted regression analyses estimated the change after the onset of the pandemic vs before the pandemic in patients’ quarterly primary care visit count; patients living in census blocks with optimal vs inadequate broadband had increased video visit use (incidence rate ratio [IRR], 1.33; 95% CI, 1.21-1.46; *P* < .001) and decreased in-person visits (IRR, 0.84; 95% CI, 0.84-0.84; *P* < .001). The increase in the rate of video visits before vs after the onset of the pandemic was greatest among patients in the lowest Area Deprivation Index category (indicating least social disadvantage) with availability of optimal vs inadequate broadband (IRR, 1.73; 95% CI, 1.42-2.09).

**Conclusions and Relevance:**

This cohort study found that patients with optimal vs inadequate broadband availability had more video-based primary care visits and fewer in-person primary care visits after the onset of the COVID-19 pandemic, suggesting that broadband availability was associated with video-based telemedicine use. Future work should assess the association of telemedicine access with clinical outcomes.

## Key Points

**Question:**

How does access to primary care in the Veterans Health Administration differ between veterans living in areas with optimal broadband internet service and veterans living in areas with suboptimal broadband internet service?

**Findings:**

This cohort study of 6 995 545 veterans seen at 937 primary care clinics providing telemedicine and in-person clinical visits found that before the COVID-19 pandemic, broadband speed was not associated with the occurrence of primary care visits. After the onset of the pandemic, for patients living in census blocks with optimal vs inadequate broadband, video visits were twice as likely to occur (4.5 vs 2.2 per 100 patients per quarter), while in-person visits were less likely to occur (13.9 vs 16.3 per 100 patients per quarter); telephone visits were similar by broadband speed category.

**Meaning:**

In this study, patients with optimal vs inadequate broadband availability had more video-based primary care visits and fewer in-person primary care visits after the onset of the pandemic, suggesting that broadband availability was associated with video-based telemedicine use.

## Teaser

This cohort study identifies disparities in primary care access in the Veterans Health Administration (VHA) based on the association between broadband service availability and primary care visit modality.

## Introduction

Telemedicine, which uses video or telephone appointments to deliver health care, has been a reality since the advent of telecommunications. However, technology has reached a stage where care can consistently be delivered in a face-to-face manner (ie, synchronous, clinician to patient) via video within the home. In addition to technological limitations and federal reticence to reimburse clinicians equally for traditional and telemedicine care, 1 potential factor associated with the slow adoption of telemedicine is patients’ disparate access to sufficient broadband speed.

The onset of the COVID-19 pandemic in March 2020 ushered in an increase in telemedicine use, owing to restrictions on in-person care and Medicare policy changes allowing equal billing for video and in-person visits as well as inaugural, but unequal, reimbursement for telephone visits.^1^ Although most of this virtual care was conducted by telephone, video visits increased rapidly.^2^ Health care systems quickly adopted telemedicine-specific video platforms. The Veterans Health Administration (VHA) has long been a pioneer in video telemedicine owing to its long-standing investments in technology and training, its ability to practice across state licensing jurisdictions, and its freedom from fee-for-service billing requirements. This foresight helped veterans transition rapidly to telemedicine after the start of the pandemic, with a near-complete substitution of virtual care for in-person care within weeks.^3^

Although many factors are associated with a patient’s use of video telemedicine (eg, age, literacy, income, rurality, and digital familiarity), adequate broadband speeds are a critical factor. Broadband encompasses wired and wireless infrastructure that determines internet connection speeds. For this study, *broadband* specifically refers to connections between the patient’s home and the VHA clinic using streaming video. According to the Federal Communications Commission (FCC), video telemedicine requires a minimum download speed of 25 MB/s and a minimum upload speed of 3 MB/s. Depending on the number of devices simultaneously streaming content, latency, and other factors, higher download and upload speeds may be needed. When combined, these factors contribute to a “digital divide,” or the gulf between those who do and those who do not have ready internet availability and internet-capable devices. The VHA has attempted to bridge this divide by supplying internet-connected devices to patients directly^4^ and negotiating with telecommunication companies to provide free unlimited data to veterans while using video telemedicine with a VHA clinician.^5^ Despite these efforts, it is estimated that 15% of veterans lack home internet and that 30% live in rural areas, limiting adequate broadband speeds for video streaming.^6^ Although the COVID-19 pandemic was an opportunity for health care systems and patients to adopt new technologies, it also highlighted and potentially exacerbated existing disparities, including those between people with and people without adequate broadband speeds.

The objective of this study was to evaluate whether access to primary care differed between veterans living in areas with optimal broadband speeds and veterans living in areas with suboptimal broadband speeds. We hypothesized that, in the transition from in-person care to telemedicine at the onset of the COVID-19 pandemic, patients residing in areas with suboptimal broadband speed would have lower use of video telemedicine. In these areas of suboptimal broadband speed, we further hypothesized that telephone care would supersede video and that overall primary care visits would be reduced. Identifying veterans with a measurable disparity in broadband availability will help evaluate the uneven distribution of access to and use of telemedicine in primary care.

## Methods

### Study Design

This is a difference-in-differences study using a cohort of veterans with primary care outpatient visits in the VHA from October 1, 2016, to June 30, 2021. This study followed the Strengthening the Reporting of Observational Studies in Epidemiology (STROBE) reporting guideline.^7^ The study was approved by the University of Iowa institutional review board and the Iowa City Veterans Affairs Healthcare System Research and Development Committee. This study was conducted without direct patient contact using data routinely collected in the electronic health record and was deemed to be of minimal risk; therefore, a waiver of informed consent was obtained.

### Data Sources

Data were obtained from the Veterans Informatics and Computing Infrastructure, an integrated system including all VHA electronic health record and administrative data. Patient-level data, including demographic characteristics and date and delivery method of outpatient primary care appointments, were obtained from the Corporate Data Warehouse. The 2010 Census Bureau census block TIGER/Line shapefile contains geographic entity codes, which were spatially merged with the fiscal year–specific latitude and longitude of each veteran’s home address to identify their census block group–based Area Deprivation Index (ADI), a measure of neighborhood socioeconomic disadvantage,^8^ and census block–based broadband availability using FCC Fixed Broadband data,^9^ which exclude mobile broadband services.

### Patient Population

The study cohort included all veterans with an outpatient primary care visit in the VHA during the study period. All primary care appointments, regardless of modality (ie, in-person, telephone, or video), were included. Care received at residential rehabilitation centers, nursing homes, or domiciliary appointments was excluded.

### Broadband Speed

Our primary exposure included broadband speeds at the census block of the patient’s residence, categorized as the availability of (1) inadequate (download speed, ≤25 MB/s; upload speed, ≤3 MB/s), (2) adequate (download speed, between ≥25 and <100 MB/s; upload speed, ≥5 and <100 MB/s), or (3) optimal (download and upload speeds, ≥100 MB/s) broadband. For each census block, the number of fixed internet providers offering broadband at or above these 3 speed thresholds was identified,^9^ and the highest broadband speed combination available was assigned to the patient’s residence. Fixed wireless internet and satellite internet were excluded because these technologies, although available virtually everywhere, have low subscription rates, high costs, and less reliability compared with other broadband technologies.

### Outcomes

Primary care clinic visits were assessed by clinic stop codes classified into mutually exclusive categories: in-person, telephone, or video-to-home visits via VA Video Connect. Once categorized, the number of primary care visits per quarter by visit modality were summed by patient, and total primary care visits were enumerated. Visits were categorized as being before (October 2016 to February 2020) or after the onset of the pandemic (March 2020 to June 2021).

### Covariates

Patient demographic characteristics included age, sex, and patient rurality, identified using the geocoded location of the patient’s home according to Rural-Urban Commuting Area codes and dichotomized into urban and rural (ie, rural, highly rural, and insular categories^10^). Differential telemedicine access has been described in the literature based on race, ethnicity, and sex.^11,12,13^ With this in evidence, these patient characteristics should be accounted for. Race and ethnicity were self-reported within the electronic health record. Race was categorized as Black or African American, other (including American Indian or Alaska Native, Asian, multiracial, or Pacific Islander), and White. Ethnicity was reported as being Hispanic, not Hispanic, or other (including missing, >1 ethnicity, or unknown).

### Statistical Analysis

We performed a difference-in-differences analysis evaluating the differences in primary care use by visit modality in the period prior to and after the onset of the COVID-19 pandemic. The dependent variable was a patient’s number of primary care visits per quarter, by visit modality from October 2016 through June 2021. Independent variables included a binary indicator for time before and after the onset of the pandemic, a categorical variable for each broadband speed category, and their interaction. The model was adjusted for patient characteristics, rurality, and quarter-year fixed effects. All regressions used the Poisson model structure with Huber-White robust SEs clustered at the census block. We report incidence rate ratios (IRRs) using a type I error rate of 0.05. We plotted quarterly visit rates by modality and overall, from October 2018 to June 2021, as well as by broadband category. All hypothesis tests were 2-sided with an a priori .05 level of significance. All analyses were conducted using Stata statistical software, version 17 (StataCorp LLC) and Microsoft SQL Server 2019 (Microsoft Corp).

We conducted subgroup analyses to evaluate whether the association between video visits and the interaction term differed across individuals based on demographic characteristics by including in the regression model the full 3-way interaction among an indicator variable for each subgroup, the postpandemic onset indicator variable, and the patient’s broadband availability category.

## Results

During the 4.5-year study period, 6 995 545 unique veterans (91.8% men; mean [SD] age, 63.9 [17.2] years; 71.9% White; and 63.0% residing in an urban area) received primary care; 38.7% lived in a census block with optimal broadband, 54.5% lived in a census block with adequate broadband, and 6.7% lived in a census block with inadequate broadband (Table 1). Veterans living in a census block with optimal broadband speed vs those living in a census block with inadequate broadband speed were younger (mean [SD] age, 62.1 [17.6] years vs 67.3 [15.1] years) and more likely to be Black (21.4% vs 8.1%), female (9.6% vs 5.6%), and live in an urban area (74.0% vs 11.2%).

**Table 1.** Study Cohort Demographic Characteristics by Broadband Speed Category and Unadjusted Difference Comparing Broadband Speed Categories Across Demographic Subgroups^a^

Covariate	No. (%)			Unadjusted difference (optimal vs inadequate broadband speed) (95% CI)
	Inadequate broadband speed (n = 473 374 [6.7%])	Adequate broadband speed (n = 3 814 697 [54.5%])	Optimal broadband speed (n = 2 707 474 [38.7%])	
Age, mean (SD)	67.3 (15.1)	64.8 (16.9)	62.1 (17.6)	−5.2 (−5.3 to −5.2)
Sex				
Female	26 360 (5.6)	286 656 (7.5)	258 924 (9.6)	4.0 (3.9 to 4.1)
Male	447 014 (94.4)	3 528 041 (92.5)	2 448 550 (90.4)	
Race				
Black or African American	38 202 (8.1)	554 971 (14.6)	579 113 (21.4)	13.3 (13.2 to 13.4)
White	387 267 (81.8)	2 846 194 (74.6)	1 820 527 (67.2)	−15.0 (−14.7 to −14.4)
Other race^b^	13 457 (2.9)	132 154 (3.5)	109 714 (4.1)	1.2 (1.2 to 1.3)
Unknown or missing	34 448 (7.3)	281 378 (7.4)	198 120 (7.3)	0.03 (−0.05 to 0.11)
Ethnicity				
Hispanic or Latino	15 054 (3.2)	240 568 (6.3)	202 519 (7.5)	4.2 (4.2 to 4.4)
Not Hispanic or Latino	434 825 (91.9)	3 387 713 (88.8)	2 383 881 (88.1)	−3.8 (−3.9 to −3.7)
Other^c^	23 495 (5.0)	186 416 (4.9)	121 074 (4.5)	−0.5 (−0.6 to −0.4)
Ever rural residence^d^	420 151 (88.8)	1 462 209 (38.3)	703 879 (26.0)	−63.8 (−62.9 to −62.6)
ADI, mean (SD)^e^	64.1 (22.0)	57.8 (25.1)	53.2 (26.4)	−10.9 (−11.0 to −10.8)

Abbreviation: ADI, Area Deprivation Index.

^a^ Broadband speeds: (1) inadequate (download speed, ≤25 MB/s; upload speed, ≤3 MB/s), (2) adequate (download speed, between ≥25 and <100 MB/s; upload speed, ≥5 and <100 MB/s), or (3) optimal (download and upload speeds, ≥100 MB/s) broadband.

^b^ Includes American Indian or Alaska Native, Asian, multiracial, or Pacific Islander.

^c^ Includes missing, more than 1 ethnicity, and unknown categories.

^d^ Includes patients who lived in a rural area at any time during the study period.

^e^ The ADI ranks neighborhoods by socioeconomic disadvantage on a scale of 0 to 100, with lower rankings indicating less social disadvantage.

**Figure 1.**
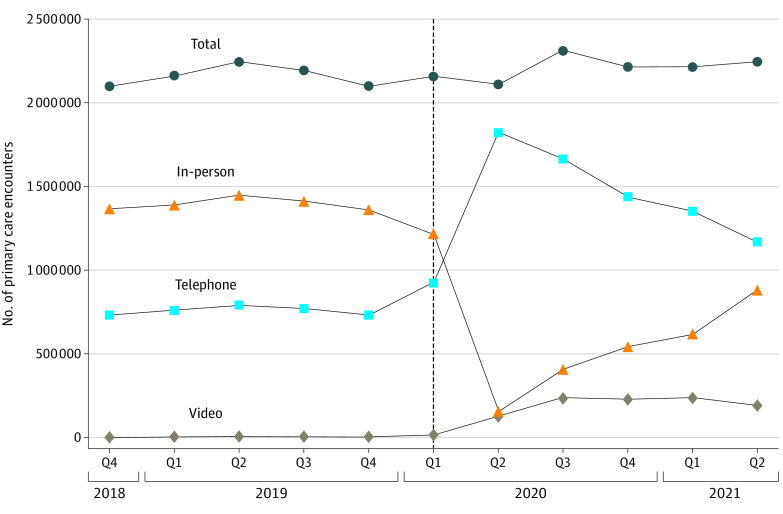
Primary Care Quarterly Visit Rate per 100 Patients, Total and by Modality Over Time, October 2018 to June 2021 The dashed vertical line indicates the beginning of the COVID-19 pandemic. Q1 indicates first quarter; Q2, second quarter; Q3, third quarter; and Q4, fourth quarter.

**Figure 2.**
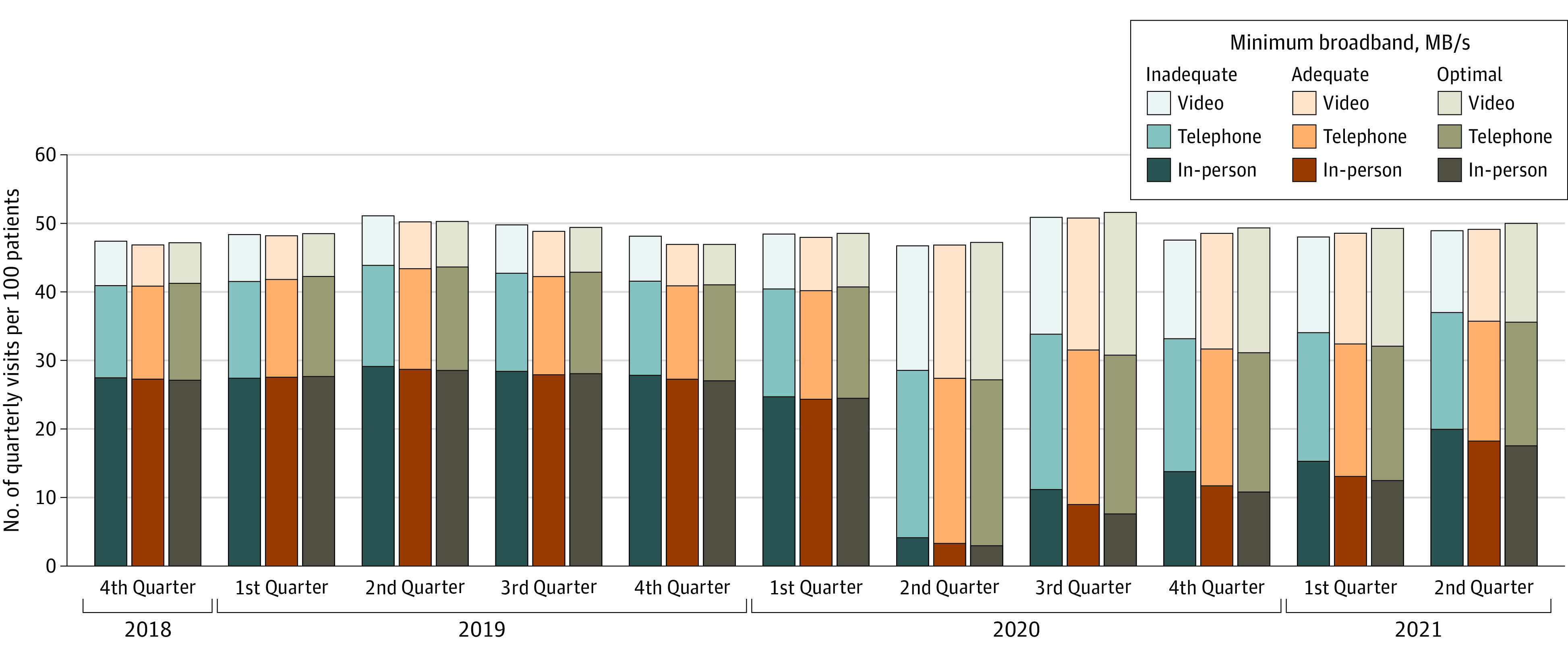
Primary Care Visits by Modality According to Census Block Access to Broadband Speed Categories Broadband speeds: inadequate (download speed, ≤25 MB/s; upload speed, ≤3 MB/s), adequate (download speed, between ≥25 and <100 MB/s; upload speed, ≥5 and <100 MB/s), or optimal (download and upload speeds, ≥100 MB/s) broadband.

Figure 1 illustrates the substitution of telephone visits for in-person visits after the onset of the pandemic, with gradual increases in video visits through June 2021. For broadband availability (Figure 2), patients in census blocks with inadequate broadband relied more on in-person and telephone visits as the pandemic progressed, whereas those in census blocks with optimal broadband speed had more video visits.

### Total Primary Care Visits

Prepandemic total primary care visits per 100 patients per quarter were similar across broadband speed categories (inadequate, 48.4; adequate, 48.0; and optimal, 48.0) (Table 2). The total number of primary care visits per 100 patients per quarter increased slightly but remained similar across broadband speed during the pandemic (inadequate, 49.3; adequate, 49.5; and optimal, 50.2). In adjusted analyses, patients living in census blocks with optimal vs inadequate broadband experienced similar changes in the rate of total primary care visits before vs after the pandemic began (IRR, 1.00; 95% CI, 0.99-1.00).

**Table 2.** Primary Care Visit Rate Before and After COVID-19 Pandemic Onset, by Visit Type and Broadband Speed Category^a^

Outcome	No. of primary care visits per 100 patients per quarter		
	Inadequate broadband speed (n = 473 374)	Adequate broadband speed (n = 3 814 697)	Optimal broadband speed (n = 2 707 474)
In-person visits			
Before COVID-19 pandemic	30.6	30.7	30.5
After COVID-19 pandemic onset	16.3	14.6	13.9
IRR (95% CI)^b^	1 [Reference]	0.89 (0.89-0.89)	0.84 (0.84-0.84)
Telephone visits			
Before COVID-19 pandemic	17.3	17.1	17.3
After COVID-19 pandemic onset	30.4	31.1	31.6
IRR (95% CI)	1 [Reference]	1.04 (1.03-1.04)	1.02 (1.01-1.02)
Video visits			
Before COVID-19 pandemic	0.03	0.04	0.05
After COVID-19 pandemic onset	2.2	3.5	4.5
IRR (95% CI)	1 [Reference]	1.19 (1.08-1.30)	1.33 (1.21-1.46)
Total primary care visits			
Before COVID-19 pandemic	48.4	48.0	48.0
After COVID-19 pandemic onset	49.3	49.5	50.2
IRR (95% CI)	1 [Reference]	1.01 (1.00-1.01)	1.00 (0.99-1.00)

Abbreviation: IRR, incident rate ratio.

^a^ Broadband speeds: (1) inadequate (download speed, ≤25 MB/s; upload speed, ≤3 MB/s), (2) adequate (download speed, between ≥25 and <100 MB/s; upload speed, ≥5 and <100 MB/s), or (3) optimal (download and upload speeds, ≥100 MB/s) broadband.

^b^ Based on a Poisson regression model that estimated the change before vs after the onset of the COVID-19 pandemic in patients’ quarterly count of primary care visits by modality among patients living in census blocks with adequate or optimal broadband compared with the change among patients living in census blocks with inadequate broadband, with adjustment for patient and geographic covariates and quarter-year fixed effects.

### Video Primary Care Visits

Prepandemic video primary care visits per 100 patients per quarter were similar across all broadband speed categories (inadequate, 0.03; adequate, 0.04; and optimal, 0.05) and universally increased after the onset of the pandemic (inadequate, 2.2; adequate, 3.5; and optimal, 4.5) (Table 2). In adjusted analyses, patients living in census blocks with optimal vs inadequate broadband exhibited an increase in the rate of video primary care visits before vs after the onset of the pandemic (IRR, 1.33; 95% CI, 1.21-1.46); increases were less but still significant for patients living in census blocks with adequate vs inadequate broadband (IRR, 1.19; 95% CI, 1.08-1.30).

### Telephone Primary Care Visits

Telephone primary care visits per 100 patients per quarter were similar across broadband speeds before the pandemic (inadequate, 17.3; adequate, 17.1; and optimal, 17.3) and increased universally after the onset of the pandemic (inadequate, 30.4; adequate, 31.1; and optimal, 31.6) (Table 2). In adjusted analyses, patients living in census blocks with optimal vs inadequate broadband exhibited an increase in the rate of telephone primary care visits before vs after the onset of the pandemic (IRR, 1.02; 95% CI, 1.01-1.02; *P* < .001); increases were similar for patients living in census blocks with adequate vs inadequate broadband (IRR, 1.04; 95% CI, 1.03-1.04; *P* < .001).

### In-Person Primary Care Visits

In-person primary care visits per 100 patients per quarter were similar across broadband speed categories before the pandemic (inadequate, 30.6; adequate, 30.7; and optimal, 30.5) and decreased during the pandemic (inadequate, 16.3; adequate, 14.6; and optimal, 13.9) (Table 2). In adjusted analyses, patients living in census blocks with optimal vs inadequate broadband exhibited a decrease in the rate of in-person primary care visits before vs after the onset of the pandemic (IRR, 0.84; 95% CI, 0.84-0.84); decreases were similar for patients living in census blocks with adequate vs inadequate broadband (IRR, 0.89; 95% CI, 0.89-0.89).

In sensitivity analyses, we included each patient’s primary care facility (defined as the modal VHA facility a patient visited during the study period) fixed effects, adjusting for differences in telemedicine adoption at the facility level. Similar results were obtained (eTable in the Supplement).

### Subgroup Analysis

In subgroup analysis, we found that video visits increased across all patient characteristics during the COVID-19 pandemic (Table 3). The largest gains in rates of video visits per 100 patients per quarter were seen among younger (age 53-67 years: before COVID-19 pandemic, 0.06%; during COVID-19 pandemic, 5.0%), female (before COVID-19 pandemic, 0.07%; during COVID-19 pandemic, 5.4%), Black or African American (before COVID-19 pandemic, 0.06%; during COVID-19 pandemic, 5.1%), and Hispanic (before COVID-19 pandemic, 0.05%; during COVID-19 pandemic, 5.8%) veterans. Those in the lowest ADI tertile (indicating least social disadvantage) had the largest gains in rates of video visits across all ADI categories (before COVID-19 pandemic, 0.04%; during COVID-19 pandemic, 4.3%). In adjusted analyses, patients in the lowest ADI category living in census blocks with optimal vs inadequate broadband exhibited an increase in the rate of video visits before vs after the onset of the pandemic (IRR, 1.73; 95% CI, 1.42-2.09), as did patients in the lowest ADI category living in census blocks with adequate vs inadequate broadband (IRR, 1.52; 95% CI, 1.25-1.84).

**Table 3.** Association Between Patient Demographic Characteristics and the Quarterly Video Primary Care Visit Rate per 100 Patients

Covariate	Video visits per 100 patients per quarter, %		IRR (95% CI)^a^	
	Before COVID-19 pandemic	During COVID-19 pandemic	Patients with adequate vs inadequate broadband speed^b^	Patients with optimal vs inadequate broadband speed^b^
Age, quartile, y				
1 (18-52)	0.05	5.0	1.13 (0.97-1.28)	1.05 (0.91-1.21)
2 (53-67)	0.06	5.0	1.18 (1.01-1.38)	1.34 (1.14-1.57)
3 (68-75)	0.04	3.6	1.38 (1.14-1.67)	1.47 (1.21-1.78)
4 (≥76)	0.02	1.8	1.16 (0.92-1.45)	1.14 (0.94-1.43)
Sex				
Female	0.07	5.4	0.89 (0.71-1.12)	1.04 (0.83-1.31)
Male	0.04	3.7	1.23 (1.12-1.36)	1.39 (1.25-1.53)
Race				
Black or African American	0.06	5.1	1.14 (0.74-1.85)	1.24 (0.81-1.90)
White	0.04	3.6	1.18 (1.06-1.30)	1.32 (1.19-1.46)
Other^c^	0.05	4.5	1.15 (0.66-2.04)	1.19 (0.67-2.12)
Ethnicity				
Hispanic or Latino	0.05	5.8	1.04 (0.69-1.57)	0.98 (0.65-1.47)
Not Hispanic or Latino	0.04	3.8	1.20 (1.08-1.32)	1.36 (1.23-1.50)
Area Deprivation Index, tertile				
1 (0-44)	0.04	4.3	1.52 (1.25-1.84)	1.73 (1.42-2.09)
2 (45-71)	0.05	3.7	1.16 (1.03-1.32)	1.28 (1.13-1.46)
3 (72-100)	0.04	3.5	1.01 (0.84-1.20)	1.09 (0.91-1.31)

Abbreviation: IRR, incident rate ratio.

^a^ Based on a Poisson regression model that estimated the change before vs after the onset of the COVID-19 pandemic in patients’ quarterly count of primary care visits by modality among patients living in census blocks with adequate or optimal broadband compared with the change among patients living in census blocks with inadequate broadband, with adjustment for patient and geographic covariates and quarter-year fixed effects.

^b^ Broadband speeds: (1) inadequate (download speed, ≤25 MB/s; upload speed, ≤3 MB/s), (2) adequate (download speed, between ≥25 and <100 MB/s; upload speed, ≥5 and <100 MB/s), or (3) optimal (download and upload speeds, ≥100 MB/s) broadband.

^c^ Includes American Indian or Alaska Native, Asian, multiracial, or Pacific Islander.

## Discussion

When the COVID-19 pandemic began, the VHA transitioned nearly completely to virtual care. We examined whether this change was differentially associated with primary care use among veterans based on broadband speeds. Overall, total primary care visits did not change, with telephone visits and, to a lesser extent, video visits replacing in-person visits. This finding was consistent across areas of differential broadband availability; however, veterans with optimal vs inadequate broadband participated in 1.33 times more video primary care visits, representing 16 additional video visits per 100 patients per quarter. Because the VHA intends to offer both in-person and virtual visits in an ongoing effort to optimize access to care, these findings highlight the role of area-level broadband availability in limiting virtual video care.

Prior to the pandemic, rates of in-person, telephone, and video visits were similar across broadband availability, with video telemedicine representing 0.09% of all visits. Overall, with the onset of the pandemic, video visits increased 90-fold, almost equaling in-person visits. Veterans living in census blocks with inadequate broadband, however, experienced the lowest video visit uptake, and at the onset of the pandemic, they relied most heavily on telephone visits. As clinics reopened and the pandemic progressed, veterans located within census blocks with inadequate broadband more quickly moved back to in-person visits. This finding brings up important equity issues associated with patient preferences, effects on work and caregiving, transportation barriers, and the patient-clinician shared decision-making model. Although more research is needed to assess whether these differences were associated with health outcomes, our findings highlight the role that local broadband availability plays in patients’ access to primary care and its potential equity implications.

Our findings support the expanded availability of broadband, in particular, upload and download speeds of 100 MB/s or more to better meet the growing need for high-speed connectivity for daily life.^14^ Previous pandemic research found that fewer video visits were associated with older age, African American race, needing an interpreter, Medicaid use, or living in a zip code with low broadband availability.^15^ However, video visits compared with telephone visits are associated with advantages in delivering high-quality care with improved diagnostic accuracy, fewer medication errors, more accurate decision-making, and higher patient satisfaction.^15,16,17,18^ Many factors affect broadband speed needs; however, lacking broadband access altogether (represented within the inadequate broadband group) also has implications beyond the use of video telemedicine. For example, it has been found that isolated rural areas or areas with a high percentage of Black residents have lower broadband access.^19^ Thus, if these areas with inadequate broadband persist, disparities in primary care access may occur and, for some, widen.

In our study, veterans with the least neighborhood-level social disadvantage (lowest ADI tertile) had higher rates of video visits when comparing optimal with inadequate broadband availability. However, based on broadband availability, video visits did not differ among those living in neighborhoods of lower socioeconomic status (highest ADI tertile). This population may face unique barriers and circumstances, making video visits less preferred or attainable. Nonetheless, previous research among uninsured patients and those with Medicaid has found both interest in and ability to successfully complete video visits, although internet availability was a common barrier.^20^ Despite living in areas of higher broadband availability, veterans in lower socioeconomic neighborhoods may still have limited access owing to affordability or lack of an adequate device (eg, smartphone, tablet, or computer) to connect to video visits. The VHA has attempted to address these barriers by providing tablets to eligible veterans,^4,21^ including those experiencing homelessness.^22^ Although this program offers patients the technology needed to participate in video visits, our study and others found that barriers, including age, race and ethnicity, and substance use disorder, continue to exist,^15,22,23^ indicating the potential need for targeted interventions to support individual patient needs. It is possible that other barriers to obtaining care, such as employment, lack of paid time off, and transportation, among others, outweigh any gains that access to a telemedicine visit might alleviate. Future work should focus on why patients choose video telemedicine, as well as the barriers and facilitators to patient preference, exploring ADI factors specifically.

Adding the context of broadband availability is an innovative way of applying publicly available data to support health care systems and clinicians planning for telemedicine expansions. For example, it is known that veterans living in rural areas travel farther distances to receive care, which can exacerbate barriers to access.^24,25,26,27,28^ We found that broadband availability and rural residence were highly correlated. Although telemedicine is viewed as a tool to overcome geographic barriers and improve access in rural areas, this is not necessarily true if underlying broadband infrastructure is not available. Identifying ways in which health care systems can bridge these gaps is an important step for telemedicine to reach its potential.

### Limitations

There are several limitations to this study that may motivate future work. First, it does not differentiate between outpatient visits that began with video telemedicine and transitioned to telephone owing to technology challenges. Understanding barriers to video telemedicine, when preferred by patients, should be considered for future implementation efforts. Second, we did not account for variations in reliance or demand for telemedicine in different VHA markets, physician familiarity with video telemedicine, or patients’ acute or chronic conditions. These factors could also be associated with telemedicine uptake to meet patient needs and should be considered. Third, our assessment of broadband availability did not include mobile telephone service coverage, which has historically been reported based on coverage areas (instead of census blocks) and technology types (instead of speeds). With 5G internet, the current 15% of adults accessing the internet exclusively via smartphone may increase^29^; however, speed and stability of the internet connection may remain affected by distance from the service tower, network congestion, and weather. Relatedly, current FCC reporting of fixed broadband may overestimate broadband availability, as an internet provider must provide the reported speeds at minimally 1 location within a census block. Fourth, quality of care, patient preference, and ability to access telemedicine if desired must be considered.

## Conclusions

This cohort study found that, in the prepandemic period, video telemedicine visits represented a small fraction of visits, with no disparity by broadband speed at the census block level. However, with the advent of the COVID-19 pandemic and a rapid transition to virtual visits, we found that those living in areas with inadequate broadband speed relied more heavily on telephone visits, with a substantially lower rate of video visits. These findings quantify a health care access disparity and underscore the necessity of internet access for primary care in a digital age. At the same time, these methods can help health care systems serving broad geographic areas make access more equitable. Rural areas, especially, would benefit from telemedicine, even when there is not a pandemic restricting in-person care. Further research should investigate the factors associated with a patient’s preference for telemedicine in primary care, along with facilitators and barriers to obtaining care via their preferred mechanism.

## Notes

Supplement. **eAppendix.** Statistical Model for the Primary Analysis **eTable.** Primary Care Visit Rate Pre- and After Pandemic Onset, by Visit Type and Broadband Speed Category
