# Supplementary material for: Association of Adequacy of Broadband Internet Service With Access to Primary Care in the Veterans Health Administration Before and During the COVID-19 Pandemic
Source: JAMA Netw Open. 2022 Oct 17;5(10):e2236524. doi: 10.1001/jamanetworkopen.2022.36524 (PMC9577674; doi:10.1001/jamanetworkopen.2022.36524)
Supplement: Supplement. — eAppendix. Statistical Model for the Primary Analysis eTable. Primary Care Visit Rate Pre- and After Pandemic Onset, by Visit Type and Broadband Speed Category [file jamanetwopen-e2236524-s001.pdf]

Supplementary Online Content

O'Shea AMJ, Baum A, Haraldsson B, et al. Association of adequacy of broadband internet service with access to primary care in the Veterans Health Administration before and during the COVID-19 pandemic. *JAMA Netw Open.* 2022;5(10):e2236524.
doi:10.1001/jamanetworkopen.2022.36524

eAppendix. Statistical Model for the Primary Analysis

eTable. Primary Care Visit Rate Pre- and After Pandemic Onset, by Visit Type and Broadband Speed Category

This supplementary material has been provided by the authors to give readers additional information about their work.

© 2022 O'Shea AMJ et al. *JAMA Network Open.*

eAppendix. Statistical Model for the Primary Analysis

The primary statical model is:

$y_{it} = \alpha_1 post_t + \alpha_2 broadband_i + \alpha_3 post_t * broadband_i + \alpha_4 X_i + \alpha_5 ADI_c + \alpha_6 rural_c + \sigma_t + \varepsilon_c$

The dependent variable (y_{it}) was patient *i*'s number of primary care visits per quarter *t*, by visit modality. Independent variables included a binary indicator for time before and after pandemic onset ($post_t$), a categorical variable for each broadband speed category ($broadband_i$), and their interaction. The model was adjusted for a vector of patient characteristics (X_i) including age (years), binary indicators for female, black, and Hispanic, the area deprivation index (ADI_c) of the census block (*c*), the rurality of the census block ($rural_c$), and quarter-year fixed effects (σ_t). The coefficient of interest was α_3.

© 2022 O'Shea AMJ et al. *JAMA Network Open.*

eTable. Primary Care Visit Rate Pre- and After Pandemic Onset, by Visit Type and Broadband Speed Category

IRR *	Inadequate[1] Broadband (N = 473,374)	Adequate[2] Broadband (N = 3,814,697)	Optimal[3] Broadband (N = 2,707,474)
In Person Visits			
Primary analysis[4]	*Reference category*	0.89 (0.89 to 0.89)	0.84 (0.84 to 0.84)
FE for PC facility[5]		0.89 (0.89 to 0.89)	0.84 (0.83 to 0.84)
Zero-inflated negative binomial[6]		0.90 (0.89 to 0.91)	0.84 (0.83 to 0.85)
Clustered errors by individual[7]		0.89 (0.89 to 0.90)	0.84 (0.84 to 0.84)
Telephone Visits			
Primary analysis[4]	*Reference category*	1.04 (1.03 to 1.04)	1.02 (1.01 to 1.02)
FE for PC facility[5]		1.04 (1.03 to 1.04)	1.02 (1.02 to 1.03)
Zero-inflated negative binomial[6]		1.04 (1.02 to 1.06)	1.03 (1.01 to 1.05)
Clustered errors by individual[7]		1.04 (1.04 to 1.04)	1.02 (1.02 to 1.02)
Video Visits			
Primary analysis[4]	*Reference category*	1.19 (1.08 to 1.30)	1.33 (1.21 to 1.46)
FE for PC facility[5]		1.20 (1.08 to 1.32)	1.34 (1.21 to 1.49)
Zero-inflated negative binomial[6]		1.18 (1.07 to 1.32)	1.32 (1.20 to 1.47)
Clustered errors by individual[7]		1.19 (1.10 to 1.28)	1.34 (1.23 to 1.45)
Total Primary Care Visits			
Primary analysis[4]	*Reference category*	1.01 (1.00 to 1.01)	1.00 (0.99 to 1.00)
FE for PC facility[5]		1.00 (1.00 to 1.01)	1.00 (1.00 to 1.01)
Zero-inflated negative binomial[6]		1.01 (1.00 to 1.02)	1.01 (1.00 to 1.02)
Clustered errors by individual[7]		1.01 (1.01 to 1.01)	1.00 (0.99 to 1.00)

1. Inadequate broadbands speeds are those ≤25/3 Mbps
2. Adequate broadbands speeds are those ≥25/5 and <100/100 Mbps
3. Optimal broadbands speeds are those ≥100/100 Mbps
4. IRR (incident rate ratio) is based on a Poisson regression model that estimated the change pre- versus after the onset of the pandemic in patients' quarterly count of PC visits by type among patients living in census blocks with 25/5 or 100/100 broadband, compared to the change among patients living in census blocks with 25/3 or less broadband, with adjustment for patient and geographic covariates and quarter-year fixed effects and robust standard errors clustered at the census block.
5. The primary analysis model, but with a fixed effect (FE) for each patient's primary care facility (defined as the modal VHA facility a patient visited during the study period)
6. The primary analysis, but using a zero-inflated negative binomial model rather than a Poisson model
7. The primary analysis, but with robust standard errors clustered at the individual level rather than at the census block.

© 2022 O'Shea AMJ et al. *JAMA Network Open.*
